# Arrhythmias in Pediatric Age: A Narrative Review

**DOI:** 10.3390/children12121580

**Published:** 2025-11-21

**Authors:** Antonio Strangio, Jessica Ielapi, Jolanda Sabatino, Rosalba De Sarro, Assunta Di Costanzo, Martina Sportelli, Federico Sicilia, Giuseppe Panuccio, Nadia Salerno, Sabato Sorrentino, Salvatore De Rosa, Daniele Torella

**Affiliations:** 1 Department of Experimental and Clinical Medicine, Magna Graecia University, 88100 Catanzaro, Italy; 2 Research Center for Cardiovascular Science, Magna Graecia University, 88100 Catanzaro, Italy; 3 Division of Cardiology, Magna Graecia University, Viale Europa, 88100 Catanzaro, Italy; 4 Pediatric Research Institute (IRP) ‘Città della Speranza’, 35127 Padua, Italy; 5 Department of Women’s and Children’s Health, University Hospital Padua, 35128 Padua, Italy; 6 Department of Medical and Surgical Sciences, Magna Graecia University, 88100 Catanzaro, Italy

**Keywords:** pediatric cardiology, arrhythmias, bradyarrhythmias, tachyarrhythmias, congenital heart disease

## Abstract

Cardiac arrhythmias in the pediatric population represent both diagnostic and therapeutic challenges due to their heterogeneous etiologies, age-dependent clinical presentations, and the variety of management strategies required. Although many electrophysiological mechanisms are shared with adults, several arrhythmias are unique to infancy and adolescence or are associated with congenital heart disease and inherited channelopathies. Clinical presentation varies considerably with age and may be unclear or nonspecific, especially in neonates and infants. In some cases, arrhythmias are benign and self-limiting; in others, they may represent the earliest manifestation of an underlying cardiac disorder, potentially progressing to hemodynamic instability, serious arrhythmic events, or sudden cardiac death. Recent advancements in electrophysiology, catheter ablation, and device technology have broadened the therapeutic landscape, improving outcomes even in neonatal and infant patients. This review aims to summarize current knowledge on pediatric arrhythmias, with a focus on clinical recognition, diagnostic evaluation, and personalized treatment strategies.

## 1. Introduction

Rhythm disorders represent a frequent cause for pediatric emergency department admission, with an estimated incidence of 55 cases per 100,000 visits [1,2,3].

The most observed arrhythmia in children is sinus tachycardia, which remains by far the most frequently reported [4]. Supraventricular tachycardias (SVTs) follow, accounting for approximately 13% of cases [3,5], while bradyarrhythmias are comparatively less frequent [6]. Among SVTs, atrioventricular re-entrant tachycardia (AVRT) represents over 70% of cases and is particularly prevalent in neonates and infants [7]. Ventricular tachycardias (VTs) although less common, are associated with higher clinical risk [8].

The incidence of arrhythmias is significantly higher in patients with congenital heart disease (CHD), primarily due to factors such as volume overload, structural abnormalities, and postoperative scars [9,10]. These patients are particularly susceptible to a wide spectrum of rhythm disorders, including both tachyarrhythmias, especially SVTs such as atrial flutter (AFL), and bradyarrhythmias like atrioventricular blocks (AVBs) and sinus node dysfunction [10,11].

Early diagnosis is crucial in the pediatric population to enable prompt management, reduce the risk of complications, and improve long-term outcomes [8,12,13,14]. Given the complexity and heterogeneity of arrhythmic presentations in children, a structured diagnostic strategy is required.

12-lead electrocardiogram (ECG) and Holter ECG monitoring [12] are key tools for diagnosis, risk stratification and follow-up planning. In addition, cardiac imaging is crucial for identifying underlying cardiac conditions, such as congenital defects, mitral valve prolapse, cardiomyopathies, coronary abnormalities, myocarditis, or cardiac mass [15].

Effective management requires a personalized approach based on arrhythmia type, patient age, comorbidities, and the overall arrhythmic risk profile [13,14]. This review provides an overview of pediatric arrhythmias, emphasizing clinical recognition, diagnostic evaluation, and evidence-based treatment approaches tailored to specific clinical scenarios.

## 2. Methods

In preparation of this narrative review, a comprehensive search of biomedical literature was conducted using the MEDLINE database, peer-reviewed life sciences journals, and online academic resources. The final search was performed in August 2025 and included only English-language international publications. The PubMed search utilized the following keywords: ‘Pediatric bradyarrhythmias,’ ‘Pediatric permanent pacing,’ ‘Pediatric supraventricular arrhythmias,’ ‘Pediatric ventricular arrhythmias,’ and ‘Ablative treatment.’ Abstracts were screened for relevance to the review topic. Key findings from selected studies were then synthesized and integrated into this manuscript. Due to the narrative review format, a formal systematic documentation of the search strategy was not required.

## 3. Bradyarrhythmias

Bradyarrhythmias in the pediatric population comprise a spectrum of disorders characterized by a heart rate below the age-specific lower limit of normal [16]. Table 1 provides reference values for resting heart rates across different pediatric age groups. The incidence of pediatric bradyarrhythmias varies significantly, depending on the underlying etiology and age group [17]. Congenital atrioventricular block (CAVB), which is often associated with maternal autoimmune—particularly anti-Ro/SSA and anti-La/SSB antibodies—and infective disease, has an estimated incidence of 1 in 15,000 to 20,000 live births [18]. Acquired forms, particularly postoperative AVB, have reported incidence rates ranging from 1% to 5%, depending on the surgical complexity and the anatomical proximity to cardiac conduction system [19]. Atrial surgery, in particular transposition of the great arteries correction via Mustard or Senning procedure, leads to sinus node dysfunction in around half of the patients [20]. Otherwise, high degree AVB is mainly associated with intervention of valve repair or replacement, atrioventricular canal surgery, and ventricular septal defect correction [21]. Furthermore, conduction system’s dysfunction can be related to several genetic mutations in the context of complex syndromes, such as Holt-Oram syndrome, which is caused by mutation in TBX5 gene and may present first-degree AVB or sinus bradycardia [22].

Considering the etiology, pediatric bradyarrhythmias may be grouped into congenital, including maternal autoimmune diseases or genetic syndromes, and acquired forms, secondary to cardiac surgery, myocarditis, or progressive degenerative diseases interesting the conduction system [17,28].

The management of pediatric bradyarrhythmias may be tailored considering the severity of rhythm disturbance and symptoms. In case of hemodynamic instability, a rapid and stepwise approach should be followed, as suggested by Pediatric Advanced Life Support Guidelines [29]. Initial treatment has to focus on optimizing oxygenation and ventilation, as hypoxia represents the most frequent reversible cause [29]. If severe bradycardia persists accompanied by signs of hypoperfusion, chest compressions should be initiated promptly, with the use of epinephrine (at a dose of 0.01 mg/kg intravenously or intraosseously, repeated every 3–5 min if necessary), in case of poor perfusion signs persistence [29]. In cases of bradycardias related to increased vagal tone or cholinergic drug toxicity, the administration of atropine at a dose of 0.02 mg/kg intravenously or intraosseously is considered an appropriate therapeutic option [29].

If hypoperfusion condition persists despite the administered medical therapy, the use of temporary pacing should be considered, especially in case of high-degree AVB or severe sinus node dysfunction [29]. In these cases, it will be necessary to consider the technical difficulties related to the procedure given the small anatomical structures and the potential cardiac variations present [30,31].

Permanent pacing is often required at some point in the lives of patients with CAVB. Determining the optimal timing for PM implant is essential, as patient body surface area influences device selection, procedural planning and complications rate. This decision should be tailored to individual risk profiles. There is broad consensus that permanent pacing is indicated in patients with high-grade or complete AVB who are symptomatic or present with risk factors such as persistently low ventricular rates (<50 bpm during daytime), pauses exceeding three times the basic cycle length, QRS duration > 120 ms, prolonged corrected QT interval, or complex ventricular ectopy [32,33]. In selected cases, pacing may also be considered in asymptomatic patients without overt risk factors; however, a cautious clinical follow-up is generally preferred, with implantation typically deferred until the patient reaches an appropriate body size. Additionally, postoperative AVB persisting for seven days or longer is recognized as a clear indication for permanent pacemaker implantation [33]. Pediatric pacing systems include both transvenous and epicardial approaches. Transvenous pacemakers (TVPMs) are generally the first-line option for older children with larger body surface areas and structurally normal hearts. In contrast, epicardial systems are typically preferred in younger patients and in those with lower body weight (<15 kg) or with structural cardiovascular abnormalities, particularly involving the vasculature. Younger age has been associated with a higher complication rate during follow-up, regardless of the pacing method [34].

Transvenous systems can cause acute although rare complications, including venous obstruction, pneumothorax, hemothorax, cardiac perforation and tricuspid valve injury, in addition to mid- long-term issues especially related to the pocket, such as hematoma and infections, and to the possible failure of the leads [35]. Epicardial leads often exhibit limited durability due to lead failure and exit block, resulting in the need for multiple surgical revisions [36]. Infection represents one of the most severe complications affecting both types of devices, given that it may progress to endocarditis, bacteremia or life-threatening sepsis [37,38]. Furthermore, in the decision-making process regarding the implantation of a permanent PM in pediatric patients, it is essential to consider the long-term need for repeated replacements of both the device and the leads, which may increase the risk of complications and pose greater procedural challenges due to the progressive loss of venous access and viable myocardial sites for effective pacing [36]. In this context, leadless pacing systems are emerging as alternative treatment options, particularly for patients with limited vascular access or those requiring long-term vascular preservation. The main advantage of leadless pacemakers (LPMs) is the significant reduction in complications compared with TVPMs, as they prevent issues related to leads and pockets, including infection, hematoma, pneumothorax, lead fracture, and lead dislocation. On the other hand, LPMs are associated with a higher risk of vascular complications and pericardial effusion compared with TVPMs. The outer sheath of currently available LPMs is 27F (9 mm), which requires the diameter of the vein used for vascular access to be at least 10 mm.

Another major limiting factor for LPMs in children is the small size of the right ventricle, which must be large enough to accommodate the leadless device. Based on current evidence, the sizes of both the peripheral veins and the right ventricle appear sufficient to permit the placement of an LPM in children weighing around 10 kg.

However, further studies are needed to support the broader adoption of this technology in daily clinical practice in this population [39].

## 4. Tachyarrhythmias

**Figure 1:**
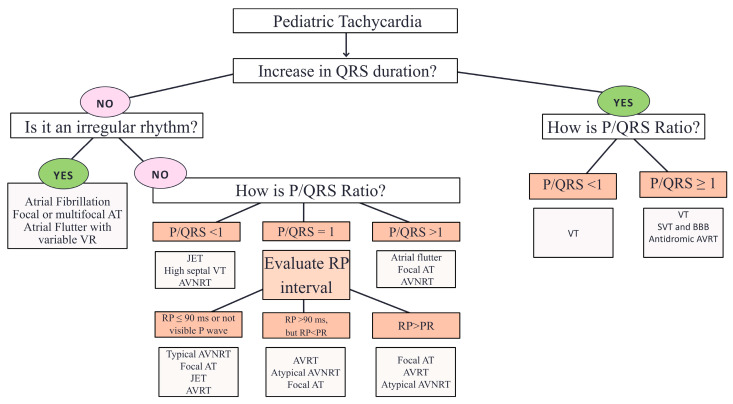
Diagnostic algorithm for tachyarrhythmias in pediatric patients. AT: atrial tachycardia. BBB: bundle branch block. JET: junctional ectopic tachycardia. AVNRT: atrioventricular nodal re-entrant tachycardia. AVRT: atrioventricular re-entry tachycardia. SVT: supraventricular tachycardia. VR: ventricular response. VT: ventricular tachycardia.

The term tachycardia refers to a heart rate that exceeds the established normal limits. It should be used regardless of whether the rate increase occurs in a single chamber—either the atrium or the ventricle—or in both. Unlike in adults, normal heart rate values in the pediatric population vary considerably with age, see Table 1. A diagnostic algorithm for tachyarrhythmias in pediatric patients is shown in Figure 1.

### 4.1. Supraventricular Tachycardias

Supraventricular tachycardias represent the most prevalent type of arrhythmia in pediatric patients and may occur at any age during childhood [40]. The incidence increases progressively from infancy through adolescence, reaching approximately 1.3 cases per 1000 patients per year by the age of 15 [41,42]. A bimodal distribution has been observed, with incidence peaks occurring between the ages of 6–9 years and again during adolescence [43].

Electrophysiologically, SVTs originate at or above the His bundle and are mediated by re-entry circuits, abnormal automaticity, or triggered activity (Table 2). Supraventricular tachycardias are categorized into three main types: re-entrant tachycardias involving an accessory pathway (AP), re-entrant tachycardias without AP, and automatic tachycardias arising from ectopic atrial foci [44].

Atrioventricular re-entrant tachycardia is the most common SVT in infants and young children. During fetal life and the first year of life, 80% of SVTs are associated with Wolff-Parkinson-White (WPW) syndrome, with the prevalence decreasing to 65% in children older than 10 years [45]. Atrioventricular re-entrant tachycardia is characterized by a circuit composed of two limbs with different conduction velocities and refractory periods: the atrioventricular node (AVN) His-Purkinje system and an AP [46]. Accessory pathways are anomalous AV connections, typically located along the AV ring, that conduct rapidly and non-decrementally due to their sodium-dependent electrophysiological properties [46]. These connections arise from incomplete embryologic separation between atrial and ventricular tissue [47]. Some APs may originate from a node-like structure at their atrial insertion, historically referred to as the ‘node of Kent’. When these histologically specialized pathways connect to the AV conduction system via a bundle descending along the right parietal wall, they can give rise to what is known as ‘Mahaim physiology’, characterized by slow and decremental conduction properties. These pathways are more accurately described as atriofascicular connections. It is important to distinguish this sling of specialized conduction tissue from the classic ‘Mahaim fiber’, which refers specifically to fibers that directly connect the AVN or His bundle to the ventricular septum. From an anatomical and functional perspective, these should be more precisely termed nodoventricular and fasciculoventricular APs, respectively, to reflect their direct association with components of the specialized conduction system [13]

Depending on their conduction properties, APs can be classified as manifest, non-manifest, or concealed [48]. Manifest pathways conduct anterogradely and retrogradely, showing preexcitation on ECG, typically defined by a PR interval ≤ 120 ms, the presence of a delta wave, and a QRS duration > 120 ms [49]. Non-manifest APs do not show preexcitation, though bidirectional conduction may be present. Concealed APs conduct only retrogradely and are never associated with preexcitation [48]. Wolff-Parkinson-White syndrome is defined by the presence of a manifest AP in combination with recurrent tachyarrhythmias [50]. In these patients, AVRT accounts for 80% of arrhythmias, followed by atrial fibrillation (AF) [51]. The most feared complication is sudden cardiac death (SCD) due to AF with rapid preexcited conduction degenerating into ventricular fibrillation [51,52,53]. In this context, AV nodal blocking agents (adenosine, verapamil, beta-blockers, digoxin, or amiodarone) must be avoided, as they may enhance conduction through the AP [54]. Atrioventricular re-entrant tachycardia typically presents with a heart rate between 150 and 250 bpm. Orthodromic AVRT exhibits a narrow QRS complex due to anterograde conduction via the AVN-His–Purkinje System and retrograde conduction via the AP [55]. In contrast, antidromic AVRT presents with a wide QRS due to anterograde conduction through the AP and retrograde conduction through the AVN [56].

Atrioventricular nodal re-entrant tachycardia is relatively uncommon in young children (~20%) but represents the most frequent form of SVT in adolescents [57]. The re-entrant circuit is located within the AVN and is characterized by near-simultaneous atrial and ventricular activation in the majority of cases [58]. The underlying mechanism involves dual AV nodal pathways typically consisting of a slow pathway and a fast pathway, although in some cases two slow pathways may be present [58]. Atrioventricular nodal re-entrant tachycardia usually presents with narrow QRS and a heart rate ranging from 150 to 250 bpm. The typical slow–fast form accounts for approximately 94% of cases, while atypical variants include fast–slow and slow–slow configurations [59]. Atrial tachycardias (AT) are less frequent (~5%) and may be focal or multifocal [60]. Focal atrial tachycardia originates from a focus with abnormal automaticity outside the sinus node [61]. It can occur in children with structurally normal hearts or following surgery for CHD [62,63]. ECG features include a regular rhythm with characteristic “warm-up” and “cool-down” phases [64]. Adenosine administration may slow AV conduction, allowing better visualization of P waves to localize the tachycardia origin [65]. Atrial tachycardia, however, can also be due, although rarely, to re-entry or to triggered activity.

Multifocal atrial tachycardia (MAT) is rare and defined by multiple ectopic atrial foci resulting in an irregular rhythm with at least three distinct P-wave morphologies on ECG [66]. It typically occurs in premature neonates with bronchopulmonary dysplasia or in infants with cardiac, pulmonary, metabolic, or neurological disorders [67].

Junctional ectopic tachycardia (JET) is a rare but potentially life-threatening SVT arising from the AV junction [68]. Congenital JET presents spontaneously in neonates, even without structural heart disease [69]. Postoperative JET occurs more commonly (5–10%) following congenital heart surgery (e.g., Tetralogy of Fallot, ventricular septal defect repair) [70,71,72].

Automatic atrial tachycardias are often drug-resistant and can evolve into tachycardia-induced cardiomyopathy (TIC) in up to 22% of children [73,74].

Atrial flutter is characterized by a macro-reentrant circuit within the atrium and is rare in pediatrics [75]. It occurs more frequently in children with CHD, systemic conditions (e.g., hyperthyroidism, infections), or myocarditis [76,77]. ECG shows rapid and organized atrial activity (250–350 bpm) with variable AV conduction (2:1, 3:1, 4:1) [78].

AF is very rare in pediatric patients and is usually associated with congenital valvular disease, post-surgical status, myocarditis, pericarditis, hyperthyroidism, familial syndromes, or genetic mutations [79]. Premature atrial contractions (PACs) are early ectopic beats originating from atrial tissue and are common in pediatric practice. Although their mechanisms are not fully understood, PACs are generally benign and rarely associated with structural or functional cardiac abnormalities. Most children are asymptomatic, and PACs usually resolve spontaneously. Clinical attention is warranted only in symptomatic or high-burden cases, which may rarely lead to arrhythmia-induced cardiomyopathy or heart failure [80].

Clinically, SVTs present differently depending on age [81]. In neonates, common symptoms include irritability, restlessness, tachypnoea, and feeding difficulties. In older children and adolescents, palpitations, chest pain, dyspnea, dizziness, and syncope are more typical [82]. Rarely, SVTs may present with hemodynamic instability, including hypotension, altered mental status, and signs of shock [83]. While many SVTs resolve spontaneously during childhood, late recurrences may occur [84].

Acute management depends on hemodynamic tolerance. In unstable patients, immediate synchronized electrical cardioversion is required, starting at 0.5–1 J/kg and increasing to 2 J/kg if necessary [85]. In stable cases, vagal maneuvers (Valsalva) are the first-line approach [86,87]. In infants, immersion of the face in cold water (diving reflex) is recommended [86]. If vagal maneuvers fail, intravenous adenosine is the next step [88]. By transiently blocking AV nodal conduction, adenosine aids both termination and diagnosis [89]. For pediatric patients, an initial dose of 0.1 mg/kg (maximum 6 mg) as a rapid IV or IO bolus in a proximal vein, immediately followed by a rapid saline flush is recommended. If conversion does not occur, administer a second dose of 0.2 mg/kg (maximum second dose 12 mg), again as a rapid bolus with a saline flush [90]. It is considered safe and effective, with rapid onset and short half-life [91]. Contraindications include asthma and preexcited AF [92,93]. In refractory cases or when diagnosis is unclear, calcium channel blockers (verapamil, diltiazem) or beta-blockers (esmolol, propranolol and labetalol) may be used [94]. Synchronized cardioversion is indicated also in pharmacologic failure [90]. If wide QRS tachycardia is present, adenosine is only appropriate in the absence of preexcitation on baseline ECG. In adenosine-refractory SVT, procainamide has shown greater efficacy than amiodarone [95].

Transesophageal pacing is a valuable diagnostic and therapeutic tool for SVTs unresponsive to adenosine and vagal maneuvers in pediatric patients [96]. Through a catheter placed in the esophagus, overdrive pacing and atrial recordings enable both termination and diagnostic clarification of SVT [97].

Long-term management, including the initiation of prophylactic antiarrhythmic therapy, should be tailored to the type of arrhythmia and clinical presentation. It is important to note that most patients experience only a limited number of SVT episodes, which often resolve spontaneously with age. Pharmacologic therapy is typically reserved for recurrent episodes to protect the child until the arrhythmia naturally subsides [13]. First-line agents for AVRT and AVNRT include oral beta-blockers (e.g., propranolol, atenolol). Flecainide is effective for preventing AVRT involving an AP [98]. Evidence is limited for drug use in pediatric AF, AFL, and automatic tachycardias [99].

Digoxin and beta-blockers remain the mainstay of therapy for AFL and AF. Procainamide is used when initial treatment fails [100] (Table 3). Catheter ablation has become the treatment of choice in symptomatic or high-risk pediatric patients due to its high curative potential, superior efficacy over drug therapy, low recurrence rate, and minimal invasiveness [101]. Reported success rates range from 80% to 96% [102]. Major complications include AVB, pericardial effusion/tamponade, and thromboembolism with higher rates observed in children weighing less than 15 kg [44,102]. Cryoablation for targets near the AVN (slow pathways, septal accessory pathways) carries a lower risk of permanent AVB due to reversible tissue effects [103,104]. In patients with asymptomatic ventricular preexcitation, ablation should be considered in the presence of high-risk predictors for sudden cardiac arrest, such as a preexcited RR interval during AF or during incremental atrial pacing ≤ 250 ms, or the presence of multiple accessory pathways [44].

In conclusion, SVTs in pediatric populations require a tailored diagnostic and therapeutic approach based on current evidence and patient-specific characteristics. Multidisciplinary collaboration and continuous education are essential for optimizing outcomes.

### 4.2. Ventricular Arrhythmias

Ventricular arrhythmias in the pediatric population encompass a wide range of conditions, from benign, self-limited premature ventricular contractions (PVCs) to life-threatening sustained tachyarrhythmias.

Premature ventricular contractions are among the most common rhythm disturbances in pediatric age, observed in up to 15% of healthy children and infants [105]. These are defined as early ectopic beats originating from the ventricles and may arise from a single focus, resulting in uniform morphology (monomorphic PVCs), or from multiple sites, leading to varying QRS morphologies (polymorphic PVCs) [88]. In most cases, PVCs are benign, asymptomatic, and resolve spontaneously, especially in children without underlying heart conditions [106,107,108]. However, some children may experience symptoms such as palpitations or chest discomfort. A high PVC burden, commonly defined as more than 20% of all ventricular beats over 24 h, has been associated with TIC, a potentially reversible form of ventricular dysfunction [109,110,111].

Premature ventricular contractions are considered potentially dangerous when they occur in the context of structural heart disease, a family history of sudden cardiac death (SCD), or when their frequency increases during exercise. In these cases, further evaluation and treatment are warranted [88,110].

In asymptomatic children with normal heart function, treatment is not generally required [106,107].

However, if impaired cardiac function develops, medical therapy is usually required. For this reason, it seems prudent, after performing a baseline evaluation, to provide ongoing follow-up for children with a high PVC burden, monitoring their left ventricular ejection fraction.

Beta-blockers are considered the first-line therapy, followed by calcium channel blockers or, in select cases, class III antiarrhythmic agents like amiodarone [88]. Catheter ablation is a highly effective option, particularly when medical therapy fails, with evidence of high success rates in several studies of both younger and older pediatric cohorts [88,112,113].

Ventricular tachycardias (VTs) are uncommon in pediatric population, with an estimated incidence around 1 per 100,000 children [114]. VT is defined by the presence of three or more consecutive ventricular beats occurring at rate exceeding age-specific normal limits [115,116].

Episodes lasting more than 30 s are classified as sustained VT, whereas those of shorter duration are referred to as non-sustained VT [116].

Monomorphic VTs are characterized by ventricular beats with single QRS morphology [117]. This group includes tachycardias originating from the right ventricular outflow tract and the left posterior fascicle, which represent the most frequent idiopathic forms occurring in structurally normal hearts and are typically associated with a benign clinical course [118]. Right ventricular outflow tract tachycardia, the most common variant in young patients, is usually due to triggered automaticity involving cyclic AMP. It often responds to vagal maneuvers, beta-blockers, or calcium channel blockers [88]. Spontaneous resolution is frequently observed, especially in infants, while older children are more likely to exhibit persistent arrhythmia [119,120]. A distinct subtype of monomorphic VT is left fascicular VT, which is typically exercise- or stress-induced [118]. This arrhythmia is caused by re-entrant circuit involving conduction system; it could be misdiagnosed with SVT for its presentation with narrow QRS [121]. It is often well-tolerated with a benign prognosis, although rare cases of TIC or SCD have been reported [122]. Left fascicular VT is also known as verapamil-sensitive VT, for its good response to this treatment, so in symptomatic patients it is recommended a first medical management with use of a calcium-channel blocking agent [118,122,123].

Bundle branch re-entrant ventricular tachycardia is a rare monomorphic VT, typically presenting on ECG with a left bundle branch block (LBBB) QRS morphology [124]. Identification of this arrhythmia should prompt thorough evaluation for underlying structural or systemic conditions, as bundle branch re-entrant ventricular tachycardia is frequently associated with dilative cardiomyopathy, myotonic dystrophy, and post-cardiac valve surgery [117]. Catheter ablation is generally effective and should be considered, particularly in patients with significant symptoms [125]. However, due to the progressive nature of His–Purkinje system disease in this context, there is an increased risk of AVB over time, which may necessitate pacemaker or implantable cardiac defibrillator (ICD) implantation [126,127]. Although relatively uncommon, VT may occasionally present in an incessant form, particularly in infants with ventricular tumors [128]. Prognosis largely depends on the duration of the arrhythmic episodes, as prolonged tachycardia can lead to ventricular dysfunction and heart failure [119]. Although it may resolve spontaneously, there have also been reports of SCD [129,130].

Polymorphic VTs are rare in pediatric age, particularly in structurally normal hearts, but are associated with higher risk of hemodynamic instability and SCD [88]. These arrhythmias may be linked to inherited channelopathies, drug toxicity (e.g., digoxin), or electrolyte imbalances. Consequently, initial management should focus on identifying and addressing the underlying cause, when feasible [131]. A well-recognized form of polymorphic VT is torsades de pointes, which occurs in the context of QT interval prolongation and is characterized on ECG by a sinusoidal pattern due to continuous variation in the QRS axis [117].

Bidirectional VTs is not a real type of polymorphic VT, but it is characterized by beat-to-beat alternation of the QRS axis, obtaining two only different morphologies [117]. This form is usually associated with pathological condition, such as catecholaminergic polymorphic ventricular tachycardia (CPVT), Andersen–Tawil, digoxin toxicity, and acute myocarditis [132,133].

When evaluating pediatric ventricular arrhythmias, it is firstly critical to rule out underlying cardiac diseases such as long QT syndrome, Brugada syndrome, CPVT, or cardiomyopathies [134]. Early recognition and tailored management of ventricular arrhythmias are vital to prevent complications like TIC or, in rare cases, SCD [119,130].

A detailed personal and family history, along with a comprehensive physical examination, is essential to guide the diagnostic work-up and the treatment strategies [88]. Symptom severity can range from asymptomatic presentation to syncope or heart failure, depending on the type, duration, and burden of the arrhythmia, as well as the ventricular function [135]. Laboratory testing, including electrolytes, thyroid, troponin, and toxicology screening, is useful for identifying reversible causes. Ambulatory Holter monitoring, exercise testing, and cardiac imaging are essential tools for quantifying arrhythmia burden and detecting underlying structural heart diseases [88,117,136]. Genetic testing should be considered when an inherited condition is suspected [134]. In selected cases, electrophysiological study may be indicated to guide therapeutic decisions, including ablation or ICD implantation [117]. However, in children without structural heart disease or genetic arrhythmia syndromes, the risk of SCD is very low, and ICD use is rarely justified [88].

Treatment should be individualized based on symptoms severity, arrhythmia frequency, and ventricular function [136]. In asymptomatic children with low arrhythmic burden and preserved heart function, a watchful waiting approach remains an acceptable strategy [88,120]. In hemodynamically unstable patients, immediate synchronized cardioversion is indicated [117]. Some idiopathic VTs, such as verapamil-sensitive fascicular tachycardia, may spontaneously resolve with age; however, pharmacologic treatment is often required due to symptoms or the potential risk of TIC [136]. Although verapamil is effective acutely, long-term management may require beta-blockers or antiarrhythmics drugs, with careful monitoring for adverse effects during follow-up [13,118]. Catheter ablation is an effective option for drug-resistant or symptomatic VT, especially when associated with ventricular dysfunction [113,134,137,138]. While generally safe, the procedure carries a higher risk of complications—such as coronary artery injury or cardiac perforation—in infants due to smaller anatomical structures [113,139]. Consequently, ablation in this age group is typically reserved for life-threatening or incessant arrhythmias. In older children, treatment decisions should be individualized based on arrhythmia characteristics, response to medical therapy, and procedural risk [116,118]. The use of 3D electroanatomical mapping has significantly reduced radiation exposure and enhanced procedural safety [140,141]. Despite high success rates, recurrence is more frequent in younger patients, highlighting the importance of close follow-up and individualized treatment plans [142].

## 5. Conclusions

The management of cardiac arrhythmias in the pediatric population represents a complex clinical challenge due to their heterogeneity in presentations, associated conditions, and prognostic implications [14]. Early identification of high-risk arrhythmic phenotypes and accurate risk stratification are crucial for the prevention of serious complications [3]. A comprehensive and systematic diagnostic approach is essential, incorporating standard ECG, extended rhythm monitoring, cardiovascular imaging, stress testing, and, when indicated, invasive electrophysiological study [12]. In cases where inherited channelopathies or familial arrhythmia syndromes are suspected, genetic testing serves as a key component of risk evaluation and management [134]. Therapeutic strategies for pediatric arrhythmias must be individualized based on arrhythmia type, patient age, symptom severity, comorbidities, and overall risk profile [13]. Bradyarrhythmias are treated based on severity and clinical status [9]. Acute symptomatic cases may require airway support, medications, or temporary pacing [3]. Permanent pacing is indicated in symptomatic patients or high-degree AVB [35]. The choice of device must be carefully made, considering the various complications related to permanent PM implantation.

Supraventricular tachycardias in pediatric patients encompasses diverse mechanisms, predominantly re-entrant and automatic forms, with clinical presentation and management varying by age and arrhythmia subtype [6]. Acute treatment aims to restore hemodynamic stability, while long-term therapy depends on recurrence risk and symptoms, and may include pharmacological and catheter ablation options [87]. Ventricular arrhythmias in paediatric patients range from benign PVCs to potentially life-threatening forms [88]. While many cases resolve spontaneously, selected patients benefit from pharmacological therapy or catheter ablation with careful long-term follow-up [105]. Effective care requires specialized electrophysiology expertise and a multidisciplinary, patient-centered approach [14]. Long-term surveillance in expert centers is key to optimizing outcomes and improving quality of life [8].

## 6. Future Directions

Conduction system pacing (CSP) has demonstrated efficacy in numerous adult studies for the prevention and reversal of pacing-induced cardiomyopathy. Pediatric patients who require a high burden of ventricular pacing, particularly those with CAVB, are also at risk of developing pacing-induced cardiomyopathy. Emerging evidence suggests that CSP may be a promising strategy in the pediatric population as well, with recent reports highlighting its feasibility in this setting [143,144]. In the future, technological advancements may enable the development of novel devices that combine the benefits of CSP and leadless stimulation. Prototypes are already under development [144,145] and may have applicability in the pediatric population. Moreover, preclinical studies have reported the use of leadless, battery-free, bioresorbable pacemakers in animal models. These devices are extremely small, allowing for percutaneous injection and endovascular delivery, and may represent a potential solution for transient AVB or serve as a bridge strategy toward definitive therapy [146]. Another fascinating and promising approach may come from gene or cell therapy, through the development of so-called biological pacemakers—genuine replicas of native pacemaker cells [147]. In the management of tachyarrhythmias, the introduction of 3D electroanatomical mapping systems, contact force-sensing technology, and remote magnetic navigation has significantly improved the safety, precision, and outcomes of catheter ablation procedures. The development of smaller, more maneuverable catheters, enhanced mapping accuracy, and the emergence of novel energy sources are expected to further expand the applicability of ablative therapy in the pediatric population.

## Figures and Tables

**Table 1:** Reference ranges for resting heart rates across different pediatric age groups.

Age Range (Years)	APLS [23]/PHPLS [24] (Beats/Minute)	PALS [25] (Beats/Minute)	EPLS [26] (Beats/Minute)	PHTLS [27] (Beats/Minute)
Neonate	110–160	85–205	85–205	120–160
0–1	110–160	100–190	100–180	80–140
1–2	100–150	100–190	100–180	80–130
2–3	95–140	60–140	60–140	80–120
3–5	95–140	60–140	60–140	80–120
5–6	80–120	60–140	60–140	80–120
6–10	80–120	60–140	60–140	(60–80)–100
10–12	80–120	60–100	60–100	(60–80)–100
12–13	60–100	60–100	60–100	(60–80)–100
13–18	60–100	60–100	60–100	60–100

APLS, Advanced Paediatric Life Support; PHPLS, Pre-Hospital Paediatric Life Support; PALS, Pediatric Advanced Life Support; EPLS, European paediatric life support; PHTLS, Pre-Hospital Trauma Life Support.

**Table 2:** Supraventricular tachycardias: pathophysiology, incidence and therapeutic strategies.

SVTs	Mechanism	Incidence	Onset	Response to Adenosine	Pharmacological Treatment	Invasive Treatment
AVRT	Re-entry involving AVN and AP	More common in neonates and young children	Neonates, infants, children	Sudden interruption	Beta-blockers Verapamil/Diltiazem Flecainide/Propafenone Sotalol Amiodarone	RF or cryoablation * (first line treatment)
AVNRT	Re-entry within the AVN	Common in adolescents	More frequent >10 years	Sudden interruption	Beta-blockers Verapamil/Diltiazem Flecainide/Propafenone Sotalol	RF or cryoablation * (first line treatment)
EAT	Abnormal automaticity	Less common	Any age, more common < 3 years	Temporary reduction of heart rate	Beta-blockers Verapamil/Diltiazem Flecainide/Propafenone Sotalol Amiodarone	RF or cryoablation * (in resistant and symptomatic forms)
JET	Abnormal automaticity	Rare	Neonates (congenital) or post-op for congenital heart disease	Temporary reduction of heart rate	Amiodarone Beta-blockers Flecainide/Propafenone Ivabradine	RF * (in severe and resistant congenital forms)
AFL	Macro-reentrant atrial	Rare	Mainly in neonates with heart disease, postoperative	Persistent tachycardia with transient AVB	Beta-blockers Verapamil/Diltiazem Digoxin Amiodarone Sotalol	RF or cryoablation *
AF	Chaotic atrial activation	Very rare	Adolescents with structural heart disease	Persistent tachycardia with transient AVB	Beta-blockers Amiodarone Flecainide/Propafenone Verapamil/Diltiazem Sotalol	RF or cryoablation with pulmonary vein isolation (age > 12 years)

* Should be deferred until the child weighs more than 15 kg. AF, Atrial Fibrillation; AFL, Atrial Flutter; AP, Accessory Pathway; AVB, Atrioventricular Block; AVNRT, Atrioventricular Nodal Reentrant Tachycardia; AVRT, Atrioventricular Reentrant Tachycardia; EAT, Ectopic Atrial Tachycardia; JET, Junctional Ectopic Tachycardia; RF, Radiofrequency, SVT, Supraventricular Tachycardia.

**Table 3:** Pharmacological management of the main types of supraventricular tachycardia.

Drug	Indications	Pediatric Dosage	Contraindications
Adenosine	AVNRT, AVRT	0.1 mg/kg IV rapid bolus (max 6 mg); if ineffective, 0.2 mg/kg (max 12 mg)	Asthma; AVB; preexcited AF
Amiodarone	Refractory SVT, Wide-complex SVT	5–15 mg/kg IV over 20–60 min; maintenance 5–15 mg/kg/min	Thyroid disease; pulmonary fibrosis; hypotension; bradycardia; long QT; AVB
Beta-blockers	SVT prophylaxis, EAT, Rate control	Propranolol: 0.01–0.1 mg/kg/dose (max 1 mg in infants, 3 mg in children) Esmolol: 100–500 mcg/kg IV bolus; maintenance 50–200 mcg/kg/min Labetalol: 0.2–1 mg/kg; maintenance 0.25–3 mg/kg/h	Asthma; AVB; acute heart failure; preexcited AF
Flecainide	AVRT	1–2 mg/kg IV (max 3 mg/kg/day) over 10–30 min;	Structural heart disease *; AVB; long QT; heart failure
Propafenone	AVRT	1–2 mg/kg IV over 10–15 min; maintenance 2–10 µg/kg/min	Structural heart disease *; AVB; long QT; heart failure
Diltiazem	Rate control	Children 0.25 mg/kg IV bolus (max 20 mg); adolescents 10–20 mg IV bolus	Neonates (<1 year); AVB; hypotension; acute heart failure; use of β-blockers; preexcited AF
Verapamil	Rate control	Children 0.1–0.3 mg/kg slow IV (max 5 mg); adolescents 5–10 mg slow IV	Neonates (<1 year); AVB; hypotension; acute heart failure; use of β-blockers; preexcited AF
Procainamide	Refractory SVT Wide-complex SVT	10–15 mg/kg IV over 30–60 min; maintenance 20–80 mcg/kg/min	Lupus, QT prolongation, heart block, torsades de pointes
Sotalol	SVT prophylaxis	1–3 mg/kg slow IV (max 100 mg) over 30–60 min	Long QT, asthma, AVB, severe bradycardia, heart failure; preexcited AF
Digoxin	Rate control	20–430 mcg/kg iv; maintenance 5–10 mcg/kg/day	preexcited AF, AVB, hypertrophic cardiomyopathy

* In selected patients appears feasible and safe when guided by comprehensive imaging and clinical judgment. AF, Atrial Fibrillation; AVB, Atrioventricular Block; AVNRT, Atrioventricular Nodal Reentrant Tachycardia; AVRT, Atrioventricular Reentrant Tachycardia; EAT, Ectopic Atrial Tachycardia; SVT, Supraventricular Tachycardia.

## Data Availability

No new data were created or analyzed in this study.

## Abbreviations

The following abbreviations are used in this manuscript:

AF	Atrial Fibrillation
AFL	Atrial Flutter
AP	Accessory Pathway
AVB	Atrioventricular Block
AVN	Atrioventricular Node
AVRT	Atrioventricular re-entrant Tachycardia
AVNRT	Atrioventricular Nodal Re-entrant Tachycardia
CAVB	Congenital Atrioventricular Block
CHD	Congenital Heart Diseases
CPVT	Catecholaminergic Polymorphic Ventricular Tachycardia
CSP	Conduction System Pacing
EAT	Ectopic Atrial Tachycardia
ECG	Electrocardiogram
ICD	Implantable Cardiac Defibrillator
JET	Junctional ectopic tachycardia
MAT	Multifocal Atrial Tachycardia
PAC	Premature atrial contraction
PM	Pacemaker
PVC	Premature Ventricular Contraction
SA	Sinoatrial
SAB	Sinoatrial Block
SCD	Sudden Cardiac Death
SVT	Supraventricular Tachycardia
TIC	Tachycardia-Induced Cardiomyopathy
VT	Ventricular Tachycardia
WPW	Wolf Parkinson White
